# Potent suppression of HIV-1 cell attachment by Kudzu root extract

**DOI:** 10.1186/s12977-018-0446-x

**Published:** 2018-09-20

**Authors:** S. Mediouni, J. A. Jablonski, S. Tsuda, A. Richard, C. Kessing, M. V. Andrade, A. Biswas, Y. Even, T. Tellinghuisen, H. Choe, M. Cameron, M. Stevenson, S. T. Valente

**Affiliations:** 1 Department of Immunology and Microbiology, The Scripps Research Institute, 130 Scripps Way, 3C1, Jupiter, FL 33458 USA; 2 Department of Molecular Therapeutics, The Scripps Research Institute, Jupiter, FL USA; 3 The Botanist’s Beach Farm, Jupiter, FL USA; 4 University of Miami Miller School of Medicine, Miami, FL USA; 5 Present Address: Roche, Basel, Switzerland

## Abstract

There is a constant need to improve antiretrovirals against HIV since therapy is limited by cost, side effects and the emergence of drug resistance. Kudzu is a climbing vine from which the root extract (*Pueraria lobata*), rich in isoflavones and saponins, has long been used in traditional Chinese medicine for a variety of purposes, from weight loss to alcoholism prevention. Here we show that Kudzu root extract significantly inhibits HIV-1 entry into cell lines, primary human CD4^+^T lymphocytes and macrophages, without cell-associated toxicity. Specifically, Kudzu inhibits the initial attachment of the viral particle to the cell surface, a mechanism that depends on the envelope glycoprotein gp120 but is independent from the HIV-1 cell receptor CD4 and co-receptors CXCR4/CCR5. This activity seems selective to lentiviruses since Kudzu inhibits HIV-2 and simian immunodeficiency virus, but does not interfere with Hepatitis C, Influenza, Zika Brazil and adenovirus infection. Importantly, depending on the dose, Kudzu can act synergistically or additively with the current antiretroviral cocktails against HIV-1 and can block   viruses resistant to the fusion inhibitor Enfuvirtide. Together our results highlight Kudzu’s root extract value as a supplement to current antiretroviral therapy against HIV.

**Electronic supplementary material:**

The online version of this article (10.1186/s12977-018-0446-x) contains supplementary material, which is available to authorized users.

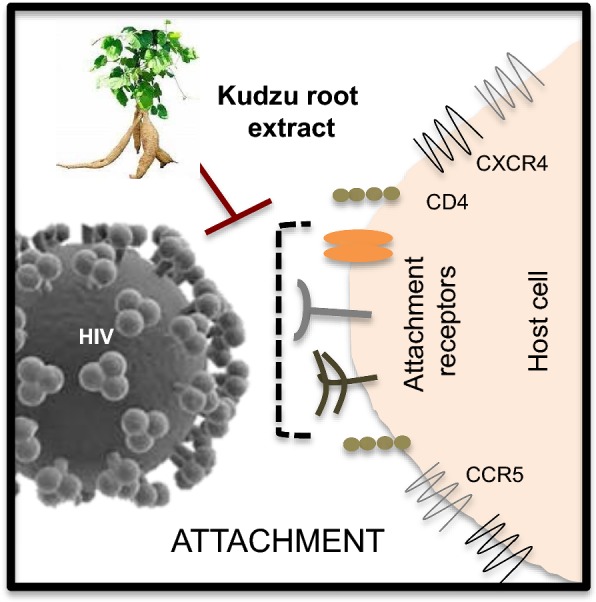

## Background

Current human immunodeficiency virus (HIV) antiretroviral therapy (ART) has dramatically benefited HIV-1 infected individuals, by reducing circulating virus to very low levels; however, it fails to completely eliminate infection and the emergence of drug resistance is an ongoing problem [1, 2]. It is highly desirable to develop additional anti-HIV-1 agents with superior efficacy and safety profiles.

HIV entry into target cells is a multistep process. The envelope glycoprotein is composed of the surface gp120 and the transmembrane gp41. The gp120 first attaches to the target cell through interactions with the negatively charged cell-surface heparan sulfate proteoglycans [3], α4β7 integrins [4, 5], the dendritic cell–specific intercellular adhesion molecular 3-grabbing non-integrin (DC-SIGN) [6] or the mannose binding C-type lectin receptors (MCLR) [7]. These attachment receptors bring gp120/gp41 into close proximity with the virus receptor CD4 and the co-receptors CXCR4 or CCR5 on the cell surface, increasing infection efficiency. Following this initial contact, interaction of gp120 with CD4 induces a conformational change in gp120, exposing the co-receptor binding site to allow interaction with the co-receptors. This also leaves gp41 in an active form, resulting in fusion of virus and cell membranes and release of the nucleocapsid into the cytoplasm [8–10]. Soon after infection, the viral population is mainly composed of R5 strains, defined by their use of the CCR5 co-receptor. As the disease progresses, the R5 strains evolve to dual X4R5 or just X4 strains, defined by their use of the CXCR4 co-receptor.

Virus attachment and fusion (collectively termed “virus entry”) have long been the target for drug development against HIV [11, 12]. Only three Food and Drug administration (FDA) approved drugs currently block the entry pathway. Enfuvirtide (Fuzeone) targets the HIV-1 envelope glycoprotein gp41 to prevent viral and host membrane fusion. It is mainly used as salvage therapy in multidrug resistance cases, given that it is dosed twice-daily by subcutaneous injection [13–15]. Maraviroc, is a selective and reversible CCR5 antagonist [16, 17], and genotypic tropism testing for co-receptor usage is usually recommended before administration. Finally, Ibalizumab is an antibody that targets the CD4 receptor. It is administrated intravenously every 2 weeks, approved for multidrug resistant cases [18]. The gp120 is critical for viral entry, but unfortunately drug discovery against this protein has remained unsuccessful so far, possibly due to the incredible ability of this protein of mutating and evading drug-selective pressure.

Natural products have always been a valuable resource for the pharmaceutical industry and have been of great benefit in virtually all-clinical therapeutic areas. Kudzu (also named *Pueraria lobata*, Kudzu vine, foot-a-night vine, vine-that-ate-the-South, and Ko-hemp) belonging to the genus Pueraria, is a fast-growing evergreen vine originating from China. Kudzu was introduced in the United States from Japan at the Philadelphia Centennial Exposition in 1876. Initially it was used as forage crop and ornamental plant, but later in the 1930s, many Southern farmers were encouraged to plant Kudzu for erosion control. Kudzu quickly became invasive, and today an estimated 2 million acres of forestland in the southern United States are covered with Kudzu.

Kudzu’s root extract (or radix Puerariae or Gegen) has been traditionally used in Asian countries (since at least 200 BC) as an herbal remedy to treat colds, headaches and diarrhea. The entire plant is edible, however the health supporting benefits of Kudzu comes from its flowers and roots, which contain many natural products including isoflavones and saponins. In Asia, Kudzu root extract is often found in food products, beverages, as well as soaps and lotions as an antimicrobial agent [19, 20]. Recently, in a clinical trial, Kudzu has been found to reduce alcoholism [21]. The chemical composition of Kudzu differs depending on geographic origin and extraction method (Additional file 1: Table 1S) [20, 22, 23]. Chemical analysis of Kudzu root extract revealed several chemical entities including Daidzein (antimicrobial and anti-inflammatory agent), Daidzin (prevents development of cancer), Genistein (anti-bacterial and anti-leukemic agent), and is also a distinct source of the isoflavone puerarin (Additional file 1: Table 1S). Kudzu root extract has also been linked to improvements in glucose metabolism, neuron regeneration, stroke prevention, inflammation, oxidative stress reduction, migraines and cluster headaches reduction [24, 25].

Given the known general antimicrobial activity of Kudzu, we investigated its activity against HIV-1 replication. Here we demonstrate that Kudzu root extract significantly inhibits HIV-1 entry into target cells. Kudzu inhibits both R5 and X4 tropic strains of HIV-1, HIV-2 and simian immunodeficiency virus (SIV). This activity seems selective since Kudzu has no activity against viruses from other families. Kudzu root extract specifically blocks the attachment of HIV gp120 to the target cell. Importantly, Kudzu acts synergistically or additively depending on the dose, with current cocktails of antiretrovirals (ARVs), and can block viruses resistant to the commercial entry inhibitor, Enfuvirtide. Overall, these results define Kudzu root extract as a potential HIV-1 inhibitor that, combined to ART, could improve therapy outcomes by acting at a novel target site of the viral replication cycle.

## Results and discussion

### Kudzu extract inhibits HIV-1 replication

**Fig. 1:**
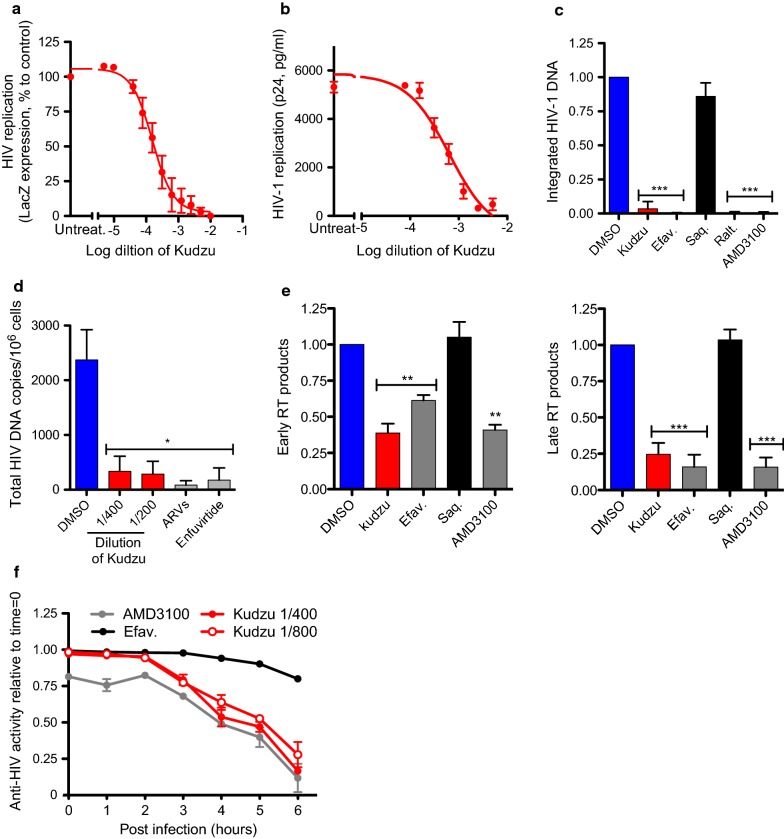
Kudzu inhibits HIV-1 replication of X4 tropic viruses. **a**, **b** Activity of Kudzu in acute infection of HeLa-CD4-LTR-LacZ cells with NL4-3. HeLa-CD4-LTR-LacZ cells were infected with HIV-1 NL4-3 strain in the presence of different dilutions of Kudzu. **a** β-Gal activity was measured 72 h later. Untreat.: untreated. The mean ± SD of 5 independent experiments is represented. **b** Viral supernatants recovered 72 h post infection were assayed for their p24 antigen content using a sandwich ELISA kit. Untreat.: untreated. Data is a mean ± SD of 2 independent experiments. **c** Impact of Kudzu on HIV-1 integration. HeLa-CD4-LTR-LacZ were infected with NL4-3 in presence of compounds for 24 h. Next day, DNA was extracted and provirus integration was quantified by Alu-PCR followed by qPCR. Saquinavir (Saq., a protease inhibitor, 200 nM), Efavirenz (Efav., a reverse transcriptase inhibitor, 200 nM), Raltegravir (Ralt., an integrase inhibitor, 200 nM), and AMD3100 (an entry inhibitor, 10 nM) were used as controls. Kudzu was used at 1:200. The mean ± SD of 5 independent experiments is represented. **d** Impact of Kudzu on HIV-1 integration in primary CD4 ^+^ T cells 24 h post-infection and treatment. A cocktail of antiretrovirals (ARVS: 180 nM AZT, a reverse transcriptase inhibitor, 100 nM Efavirenz and 200 nM Raltegravir) and Enfuvirtide (1 μg/ml) were used as controls. Shown is the mean ± SD of 3 independent experiments. **e** Activity of Kudzu on reverse-transcription products of HIV-1 10 h post-infection. HeLa-CD4-LTR-LacZ were infected with NL4-3 in presence of compounds for 10 h. Next, DNA was extracted and early and late RT products were measured by qPCR. Kudzu was used at 1:200. Error Bars from qPCR (n = 3) ± SD from 2 independent experiments for early products. The mean ± SD of 4 independent experiments is represented for late products. **f** Time-of-addition experiment of kudzu in HeLa-CD4-LTR-LacZ infected with NL4-3 strain. Kudzu or control compounds (4 nM AMD3100 and 10 nM Efavirenz) were added at 1, 2, 3, 4, 5 and 6 h postinfection. β-Gal activity was measured 72 h later. Data shown  is representative of 2 independent experiments. The two-tailed paired *t* test was used for statistical comparisons. *: *p* value < 0.05, ***: *p* value < 0.0005

HIV-1 susceptibility to Kudzu root extract was assayed using a reporter cell line that stably expresses the β-galactosidase (β-Gal) gene (LacZ) [HeLa-CD4-LTR-LacZ cells]; LacZ expression is driven by the 5′LTR promoter of HIV-1 and responds to Tat expressed by an incoming virus. HeLa-CD4-LTR-LacZ cells were infected with HIV-1 isolate NL4-3 (clade B, X4 tropic), in the presence of increasing dilutions of Kudzu, and β-Gal activity was measured 72 h later (Fig. 1a). The inhibition of Tat-dependent transcription of LacZ was dose-dependent, presenting an IC_50_ of 1:5263 ± 6 × 10^−5^ dilution of the Kudzu extract. More than 90% inhibition was observed at dilution 1:1600, a concentration that does not impact HeLa-CD4-LTR-LacZ cells viability (Additional file 1: Fig. 1SA). The IC_50_ of currently used ARVs was included for comparison (Additional file 1: Fig. 2SA).

Kudzu root extract is a tincture comprised of 33% of dried kudzu root and 66% of solvent (a mix of ethanol and glycerol). To verify that Kudzu activity was not aspecifically linked to these solvents, both solutions, at different percentages, were tested in infectivity assays (Additional file 1: Fig. 2SB and C). No viral inhibition was observed at the highest and effective dilution of Kudzu (1:200), which corresponds to 0.3% glycerol or ethanol.

The activity of Kudzu was also assessed by measuring p24 capsid in the supernatant by p24 ELISA, revealing an IC_50_ of 1:1556 (Fig. 1b). The differences in IC_50_ between the β-Gal and p24 ELISA assays most likely reflect the ability of p24 ELISA to detect all p24 production, whether the protein is incorporated into virions or not. Similar results were obtained with Kudzu purchased from another company (data not shown), suggesting that Kudzu’s activity is consistent between brands.

### Kudzu extract blocks the first steps of HIV-1 entry into target cells

To investigate the mechanism by which Kudzu suppresses HIV-1, we first monitored the integration of proviral DNA into the genome of HeLa-CD4-LTR-LacZ cells 24 h post-infection by Alu-PCR, followed by quantitative real time PCR (qPCR). Kudzu (1:200) significantly inhibited the integration of proviral HIV DNA, similar to Efavirenz (a reverse transcriptase inhibitor, 200 nM), Raltegravir (an integrase inhibitor, 200 nM) and AMD3100 (a CXCR4 antagonist, 10 nM), used as positive controls. As expected, Saquinavir, a protease inhibitor (200 nM) did not inhibit HIV integration during this 24 h assay (Fig. 1c). Dimethyl sulfoxide (DMSO) was used as negative control since the ARVs are solubilized in 0.001 or 0.002% DMSO. Furthermore, ethanol and glycerol, at the highest concentrations of Kudzu, did not interfere with HIV replication (Additional file 1: Fig. 2SB and C).

To insure Kudzu’s activity was not cell line dependent, we also assessed the activity of Kudzu on primary human CD4^+^T cells isolated from blood of 3 individuals. Cells were infected for 24 h with NL4-3 in the presence of the most potent but non-toxic dilutions of Kudzu (1:400 and 1:200; Additional file 1: Fig. 1SB), or a cocktail of ARVs (Raltegravir 200 nM, Efavirenz 100 nM and AZT 180 nM) or Enfuvirtide (1 μg/ml). Total viral DNA was measured by qPCR (Fig. 1d). Kudzu inhibited the infection of primary human CD4^+^T cells equally well as Enfuvirtide or a cocktail of ARVs. Together these results suggest that Kudzu activity is not cell type dependent and targets an early event of the HIV-1 life cycle.

We next measured the early and late HIV-1 reverse transcription (RT) products by qPCR 10 h post-infection of HeLa-CD4-LTR-LacZ cells (Fig. 1e). Efavirenz and AMD3100 (200 nM and 10 nM respectively) used as controls, inhibited early RT products by approximately 40% and 60% respectively, while the late RT products were decreased by approximately 84%, consistent with the literature [26]. Treatment of the cells with Kudzu resulted in a similar reduction of early and late RT products to controls (60% and 75% respectively). Saquinavir (200 nM), as expected, did not impact the production of late RT products. Altogether these results suggest that Kudzu inhibits an early event occurring before or at the reverse transcription step.

To further understand which step was blocked by Kudzu, we performed time-of-addition assays as previously described [27], using entry and RT inhibitors as controls. We infected HeLa-CD4-LTR-LacZ cells with NL4-3, then added Kudzu at different time points post-infection (from 1 to 6 h), and measured β-Gal activity 72 h later (Fig. 1f). Both dilutions of Kudzu (1:800 and 1:400) displayed similar inhibitory kinetics to the entry inhibitor AMD3100 (4 nM). When Kudzu or AMD3100 were added 3 h post infection, their inhibitory activity started to decline, showing almost no activity if added 6 h later. As expected, Efavirenz (10 nM) displayed stronger inhibition when added at later time points. These results suggest that Kudzu inhibits the entry step of HIV-1 into the target cell.

### Kudzu extract inhibits HIV-1 infection independently of tropism

**Fig. 2:**
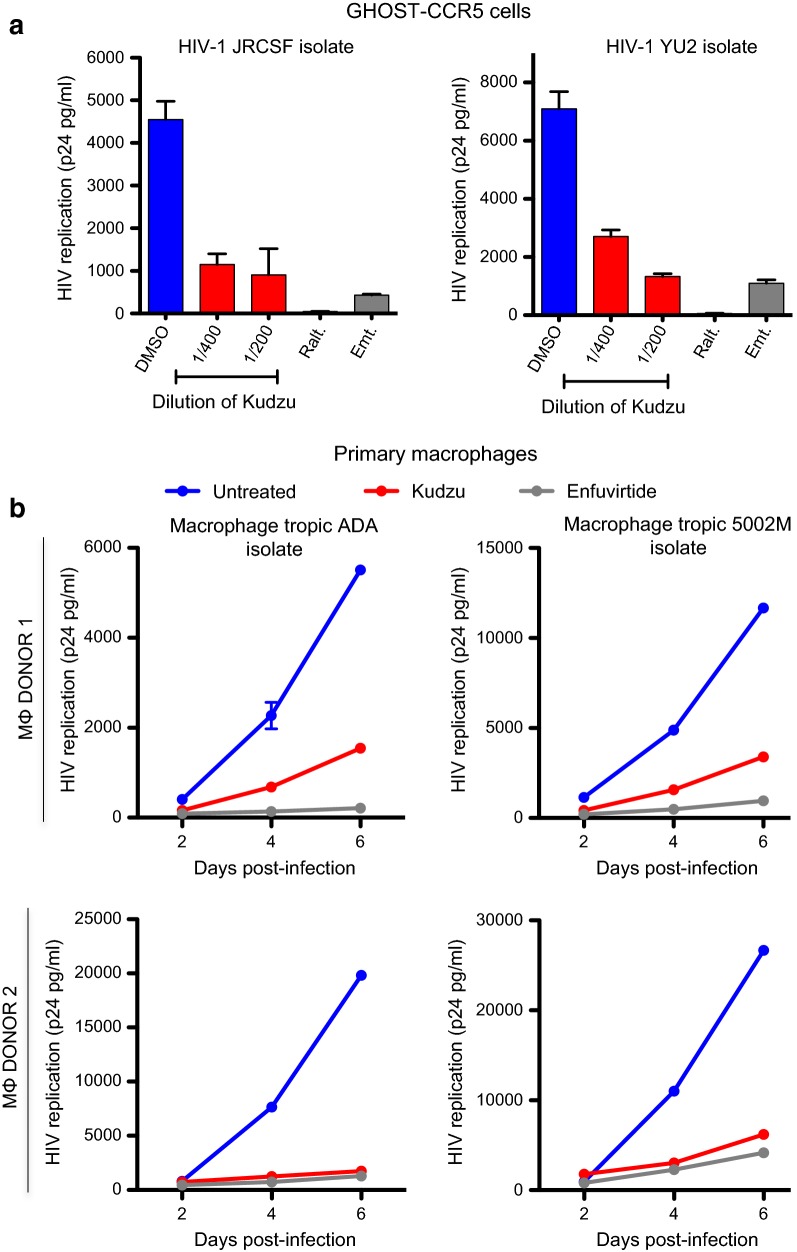
Kudzu blocks HIV-1 replication of R5 tropic viruses. **a** Activity of Kudzu on acute replication of GHOST-CCR5 cells with JRCSF and YU2 viruses. Cells were infected with both viruses in presence of 2 potent dilutions of Kudzu. Viral supernatants recovered 72 h post-infection from cells were assayed for their p24 antigen content. Raltegravir (Ralt, an integrase inhibitor, 100 nM) and Emtricitabine (Emt., a reverse transcriptase inhibitor, 100 nM) were used as controls. The mean ± SD of 2 independent experiments is shown. **b** Impact of Kudzu on acute replication of primary Human macrophages infected with macrophage-tropic stains ADA and 5002 M. Cells were infected with both viruses in presence of 1:100 dilution of Kudzu or 5 μg/ml of Enfurvirtide (a fusion inhibitor). Viral supernatants recovered at different times post-infection were assayed for their p24 antigen content

The interaction between gp120 and CD4, followed by interaction with CXCR4 or CCR5 is determinant for HIV-1 entry into the target cell. To determine if the coreceptors usage is determinant for Kudzu-mediated inhibition of HIV-1, we tested the susceptibility of R5 tropic viruses. We infected GHOST-CCR5 cells with JRCSF or YU2 viruses, in the presence of Kudzu concentrations that did not impact cell viability (1:400 and 1:200; Additional file 1: Fig. 1SC), and assessed p24 in the supernatant 72 h post infection (Fig. 2a). Raltegravir (100 nM) and Emtricitabine (a reverse transcriptase inhibitor, 100 nM) were used as positive controls. We observed a dose-dependent inhibition of both R5 viruses, suggesting that Kudzu inhibits HIV-1 entry independently of co-receptor usage.

Next, we assessed the susceptibility of Kudzu against infection of primary macrophages from two donors, using two R5 macrophages tropic viruses (ADA and 5002 M). We measured p24 production in the supernatant at different times post-infection. Kudzu (1:100) drastically inhibited HIV-1 replication of both viruses in macrophages from the two donors, with very similar kinetics as the fusion inhibitor Enfuvirtide (5 μg/ml, Fig. 2b). In sum, Kudzu seems to be able to block HIV-1 independently of tropism and cell type, important features as HIV-1 changes receptor usage during disease progression.

### Kudzu-mediated inhibition of viral entry is specific to lentiviruses

**Fig. 3:**
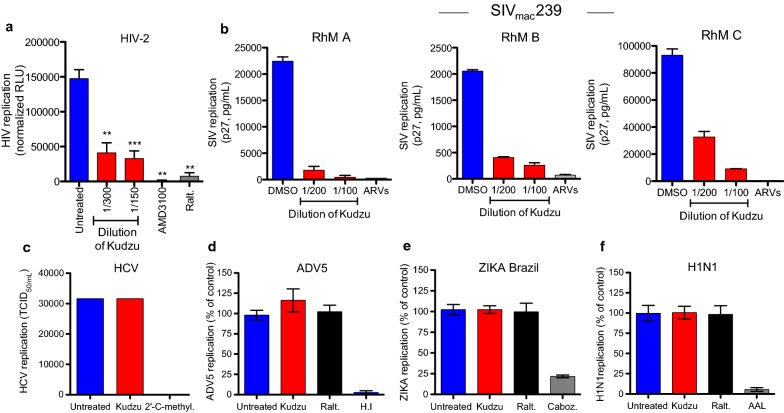
Activity of Kudzu on diverse viruses. **a** Activity of Kudzu in acute infection of TZM-bl with HIV-2 CBL-20 strain. TZM-bl cells were infected with HIV-2 in presence of different concentrations of Kudzu for 24 h. Luminescence normalized to total protein was measured 48 h later. AMD3100 and Raltegravir used at 10 nM and 100 nM respectively. Data is the mean ± SD of 3 independent experiments. **b** Activity of Kudzu on SIV-infected primary rhesus macaque cells 6 days post-infection. Virus in the supernatant measured by capsid p27 ELISA. A cocktail of antiretrovirals (ARVs, Emtracitabine, Raltegravir and Tenofovir, 200 nM) was used as control. **c** No effect of Kudzu on HCV infection in Huh 7.5 cells. 2′-Cmethyladenosine (2′-C-methyl., 10 μM) was used as a control. Results represent the mean ± SD of 2 independent experiments. **d**–**f** Hela-CD4-LTR-LacZ were infected with the indicated viruses in the presence of the indicated compounds. The cells were stained 24 h later using virus-specific antibodies to assess the levels of infection and analyzed by flow cytometry. **d** Kudzu has no activity on ADV5 virus infection of HeLa-CD4-LTR-LacZ cells. Heat inactivated virus (H.I) and Raltegravir (Ralt., 100 nM) were used as controls. Results represent the mean ± SD of 3 independent experiments. **e** No activity of Kudzu on ZIKA Brazil virus infection of HeLa-CD4-LTR-LacZ cells. Cabozantinib (Caboz., 1 μM) and Raltegravir (Ralt., 100 nM) were used as controls. Results represent the mean ± SD of 3 independent experiments. **f** No impact of Kudzu on H1N1 virus infection of HeLa-CD4-LTR-LacZ cells. Aleuria Aurantia Lectin (AAL, 100 nM) and Raltegravir (Ralt., 100 nM) were used as controls. Results represent the mean ± SD of 3 independent experiments. Kudzu was used at the dilution 1:200 in **c**–**f**. The two-tailed paired *t* test was used for statistical analysis. **: *p* value < 0.005, ***: *p* value < 0.0005

We set out to evaluate the breath of Kudzu activity against other lentiviruses such as HIV-2 and SIV or viruses from other families such as Hepatitis C virus (HCV), Influenza A (H1N1), Zika virus (Zika Brazil) and adenovirus serotype 5 (ADV5) (Fig. 3).

HIV-2, initially found in West Africa, has spread to Europe, other parts of Africa, India and the United States. HIV-2 is naturally resistant to nonnucleoside reverse transcriptase inhibitors, to some protease inhibitors [28, 29] and to Enfuvirtide [30]. We assessed Kudzu’s (1:300 and 1:150) activity against the highly cytopathic isolate HIV-2 CBL-20 strain (X4 tropic virus) [31], using TZM-bl cells expressing the luciferase gene under the control of the HIV LTR. Luminescence counts were quantified 48 h later and normalized to total protein concentration (Fig. 3a). Kudzu significantly reduced HIV-2 replication similarly to AMD3100 (10 nM) or Raltegravir (100 nM) used as controls.

Next, we investigated the effect of Kudzu on the replication of SIV (Fig. 3b). Primary macaque CD4^+^T cells from three independent rhesus macaques (RhM A, B and C) were infected with SIV_mac_239. Six days post-infection, p27 capsid in the supernatant was measured by ELISA. A cocktail of ARVs (Emtracitabine, Tenofovir and Raltegravir, 200 nM) was used as control. Kudzu extract inhibited SIV_mac_239 replication in a dose-dependent manner at concentrations (1:200 and 1:100) that did not alter cell viability (Additional file 1: Fig. 1SE). Altogether, these results suggest that Kudzu has a broad activity against lentiviruses, likely because these share similar target cell entry mechanisms.

HCV replication was investigated using Huh-7.5 cells in the presence of either Kudzu (1:200) or 2′-C-methyladenosine (10 μM), an RNA-dependent RNA polymerase inhibitor known to suppress HCV replication. Unlike the latter, Kudzu had no effect on HCV infection (Fig. 3c). The replication of H1N1, Zika or ADV5 was also assessed in HeLa-CD4-LTR-LacZ cells in the presence of Kudzu or known inhibitors of each virus. The Aleuria Aurantia lectin (100 nM) binds the envelope hemagglutinin glycoprotein of H1N1 and blocks its entry [32]. Cabozantinib (1 μM), a tyrosine-kinase inhibitor, suppresses Zika infection [33]. ADV5 infection was controlled by heat inactivation. While all our positive controls proved able to inhibit these viruses, Kudzu (1:200) showed no activity (Fig. 3d–f).

Collectively, these results suggest that Kudzu is specific to lentiviruses, HIV-1, HIV-2 and SIV. This large breath of activity suggests Kudzu targets an aspect of viral entry that is very conserved across lentiviruses, which may potentially limit resistance development.

### The anti-HIV-1 activity of Kudzu depends on gp120

**Fig. 4:**
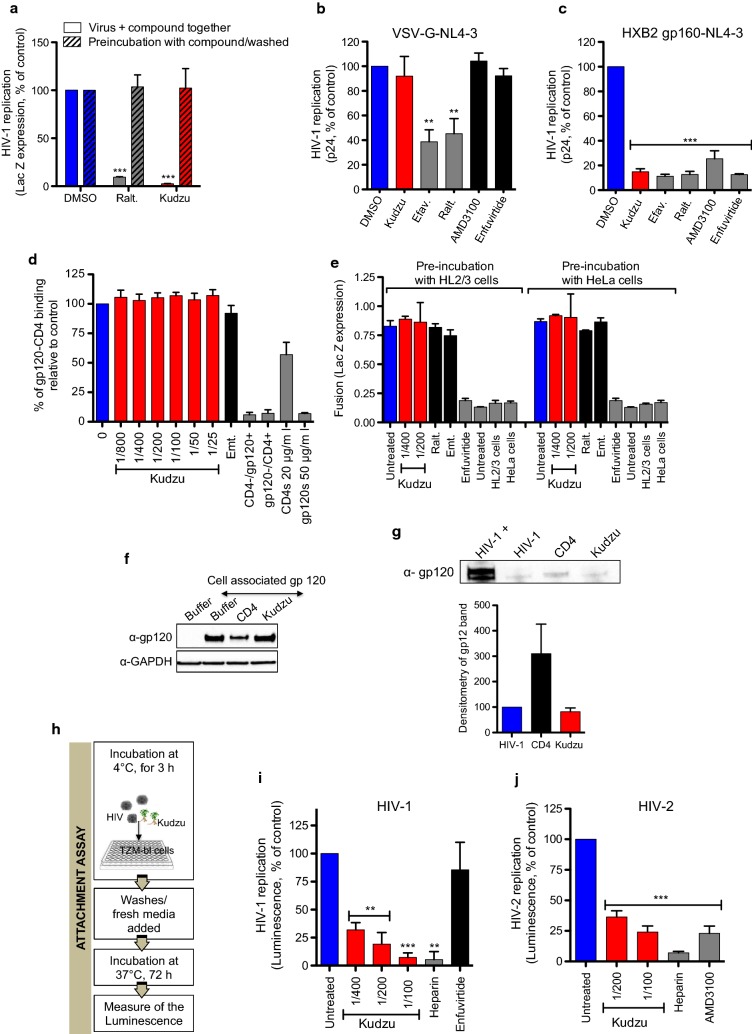
Kudzu blocks HIV-1 attachment. **a** Kudzu specifically affect the virus and not the cells. Kudzu (1:400) was either preincubated for 6 h with the HeLa-CD4-LTR-LacZ cells then washed and NL4-3 virus was added to the cells (“preincubation with compound/washed”) or simultaneously added with the virus to the cells (“virus + compound together”). β-Gal activity was measured 72 h later. Ralt.: Raltegravir, 100 nM. Results represent the mean ± SD of 3 independent experiments. **b** Absence of activity of Kudzu on infected HeLa-CD4-LTR-LacZ cells with VSV-G-NL4-3 pseudovirus. Efavirenz (Efav., 200 nM), Raltegravir (Ralt., 200 nM), AMD3100 (10 nM) and Enfuvirtide (1 μg/ml) were used as controls. Kudzu: 1:400 dilution. Viral supernatants recovered 72 h postinfection from cells were assayed for their p24 antigen content. The mean ± SD of 4 experiments is represented. **c** Activity of Kudzu on infected HeLa-CD4-LTR-LacZ cells with HXB2 gp160 pseudotyped NL4-3 env-. Same controls and Kudzu dilution as in **b**. Viral supernatants recovered 72 h post-infection from cells were assayed for their p24 antigen content. The mean ± SD of 4 independent experiments is presented. **d** No impact of Kudzu on interaction of monomeric YU2 gp120 and CD4-Ig receptor in ELISA. s: soluble. Emtricitabine (Emt., 500 nM) and soluble gp120 or CD4 were used as controls. The mean ± SD of 4 independent experiments is represented. **e** Kudzu does not affect fusion of HeLa-CD4-LTR-LacZ and HL2/3 cells. Emtricitabine (Emt., 100 nM), Enfuvirtide (1 μg/ml), cells only and untreated cells were used as controls. β-Gal activity was measured 48 h later. Data is the mean ± SD of 3 independent experiments. **f** No activity of Kudzu on shedding of transfected JRCSF gp160 in HEK293T cells, revealed by western blot. Data is representative of 3 independent experiments. CD4-Ig (45 μg) was used as a control. **g** No activity of Kudzu on shedding of NL4-3 gp120 from NL4-3 virus. HIV-1 directly loaded on gel (HIV-1+) was used as positive control of the gp120 protein. The other samples were filtered through a column and a fraction of the flow-through was analyzed by western blot. CD4 (30 μg) was used as control of the shedding.  Data is representative of 2 independent experiments and quantification of the 2 independent experiments is shown below the blot. **h**–**j** Kudzu significantly reduces the attachment of HIV-1 and HIV-2 to TZM-bl cells. **h** Schematic of the attachment assay. The attachment assay is performed by adding virus to cells in presence of the different compounds for 3 h at 4 °C. After this period of incubation at 4 °C, the cells are washed with cold PBS and then allowed to incubate at 37 °C for 72 h before luciferase is quantified. **i** Kudzu inhibits HIV-1 attachment. Enfuvirtide (a fusion inhibitor, 1 μg/ml) and Heparin (competes with Heparan sulfate proteoglycans binding to gp120, 1 mg/ml) were used as controls. The data is the mean ± SD of 3 independent experiments. **j** Kudzu inhibits HIV-2 CBL-20 attachment. AMD3100 (10 nM) and Heparin were used as controls. The data is the mean ± SD of 4 independent experiments. The two-tailed paired *t* test was used for statistical comparisons. **: *p* value < 0.005, ***: *p* value < 0.0005

The interaction between gp120 and CD4, followed by interaction with CXCR4 or CCR5 is determinant for HIV-1 entry into the target cell. After determining that Kudzu does not affect CD4, CXCR4 and CCR5 cell surface expression (Additional file 1: Fig. 3S), we investigated whether Kudzu affects the virus or the cells. As such, we either pre-incubated Kudzu with HeLa-CD4-LTR-LacZ cells for 6 h, then washed the cells and added NL4-3 virus; or, incubated kudzu and NL4-3 at the same time with HeLa-CD4-LTR-LacZ cells. β-Gal activity was measured 72 h later (Fig. 4a). Kudzu (1:400) was significantly active only when added to the cells simultaneously with the virus. Similar result was obtained with the negative control Raltegravir (100 nM). These results suggest that Kudzu mainly affects the virus, and is not the result of an activity on the target cell.

Given the key role of gp120 and gp41 proteins in the entry process, we assessed whether gp160 (precursor of gp120 and gp41) was necessary for Kudzu’s activity by comparing susceptibility of HIV virus-like particles pseudotyped with either the vesicular stomatitis virus G (VSV-G) envelope protein or the X4 gp160 envelope from HXB2 strain. We infected HeLa-CD4-LTR-LacZ cells with VSV-G-NL4-3 or HXB2 gp160-NL4-3 in the presence of Kudzu or a series of control compounds, and measured p24 production in the supernatant 72 h later (Fig. 4b and c). Efavirenz (200 nM) and Raltegravir (200 nM), which inhibit HIV-1 post-entry events, decreased both VSV-G-NL4-3 and HXB2 gp160-NL4-3 infection. However, the entry inhibitor AMD3100 (10 nM), the fusion inhibitor Enfuvirtide (1 μg/ml) and Kudzu (1:400) significantly inhibited HXB2 gp160-, but not VSV-G-driven infection of the cells. Collectively, these data suggest that Kudzu targets HIV entry, and is dependent on virus-target cell entry mechanisms mediated by gp120 and/or gp41.

To investigate whether Kudzu was targeting gp120 directly, we first assessed its ability to interfere with the interaction between gp120 and CD4. For this, we coated monomeric recombinant gp120 on an ELISA plate and measured its interaction with CD4-Ig (as in [34]), in the presence of increasing concentrations of Kudzu (Fig. 4d). The intensity of the absorbance is directly proportional to the binding of gp120 to CD4-Ig. Kudzu did not interfere with the gp120-CD4-Ig interaction, while soluble gp120 and soluble CD4 both acted as competitors. Emtricitabine (500 nM) was used as a negative control. The background of the assay was evaluated by measuring the signal generated in buffer-coated wells (instead of gp120), as well as buffer-treated wells (instead of CD4-Ig). Together these results suggest that Kudzu does not interfere with gp120 interaction with CD4.

We further investigated whether Kudzu could interfere with the fusion between gp160-expressing cells and target cells that express both CD4 and CXCR4 (Fig. 4e). HL2/3 cells express viral Gag, Env, Tat, Rev, and Nef proteins. Their co-culture with HeLa-CD4-LTR-LacZ cells results in cell fusion, and subsequent activation of 5′-LTR-driven LacZ expression by HL2/3-produced Tat protein. In this assay, we individually pre-incubated HL2/3 cells or HeLa-CD4-LTR-LacZ cells with compounds for 2 h before co-culture for another 48 h, followed by measurement of β-Gal activity. As shown in Fig. 4e, while Enfuvirtide (1 μg/ml) inhibited cell fusion, Kudzu (1:400 and 1: 200) had no inhibitory activity. Raltegravir (100 nM), Emtracitabine (100 nM), untreated and cells alone were used as negative controls. Together these results suggest that Kudzu does not interfere with the fusion between virus and cell membranes, especially with gp120 interaction with CXCR4 and gp41mediated fusion.

We then evaluated the effect of Kudzu on the shedding of gp120 (Fig. 4f), as previously described [35]. The gp120 was ectopically expressed in HEK293T cells after transfection of JR-FL gp160Δtail constructs. After 48 h, cells were incubated for 4 h with Kudzu (1:400), or soluble CD4-Ig (45 μg) used as a positive control. Cells were washed, lysed, and the remaining gp120 attached to the cell surface was detected by immunoblotting using anti-gp120 serum. As shown in Fig. 4f, the amount of gp120 remaining after Kudzu treatment was similar to the control, while soluble CD4-Ig decreased the presence of gp120 on the cell surface. To confirm this result, we exposed concentrated NL4-3 virus to Kudzu (1:200) or CD4-Ig (30 μg) for 6 h, and then passed the solution through a 300-kDa filter, to recover shedded gp120 in the flow-through. A fraction of the flow-through was then analyzed by immunoblotting with an anti-gp120 serum. The gp120 bands and corresponding densitometry are shown in Fig. 4g. Kudzu did not increase shedding of gp120 from the virus surface as opposed to CD4. Altogether these results suggest that the inhibition of HIV-1 entry by Kudzu is not mediated by gp120 shedding.

### Kudzu inhibits HIV attachment to the target cell

As we mentioned above, gp120 first attaches to the target cell by interacting with cell-surface heparan sulfate proteoglycans, α4β7 integrins, DC-SIGN or MCLR. These attachment receptors bring gp120/gp41 into close proximity to CD4 and CXCR4 or CCR5, increasing infection efficiency. As such, we monitored the involvement of Kudzu in HIV-1 attachment to the target cell, as previously described [36, 37]. Pre-chilled TZM-bl cells were incubated with NL4-3 and increasing concentrations of Kudzu for 3 h at 4 °C (Fig. 4h). This allows only attachment of the virus to the cell, but not fusion and entry of the virus. After washing with cold PBS to remove unattached virions and/or drugs bound to virions, fresh medium (without compounds) was added. The cells were then further incubated at 37 °C for 72 h, before luciferase activity was measured. The peptide fusion inhibitor Enfuvirtide (1 μg/ml), which binds to gp41, was used as a negative control, while soluble heparin (1 mg/ml), which reduces attachment of the virus to the target cell by competing with the cell surface heparan sulfate proteoglycans [38], was used as a positive control. We observed a dose-dependent inhibition of virus attachment in the presence of Kudzu compared to the untreated control (Fig. 4i). We also verified that Kudzu did not affect TZM-bl cell viability at the indicated concentrations (Additional file 1: Fig. 1SD left). Similar results were obtained with HIV-2 (Fig. 4j). Kudzu inhibited HIV-2 attachment to the same extent as the entry inhibitor AM3100, which tightly binds to CXCR4 on the cell surface. Taken together our results suggest that Kudzu root extract blocks attachment of gp120 to the cell surface, a process independent of CD4, thus inhibiting the first step of the HIV entry life cycle.

### Kudzu does not inhibit HIV-1 late replication events

**Fig. 5:**
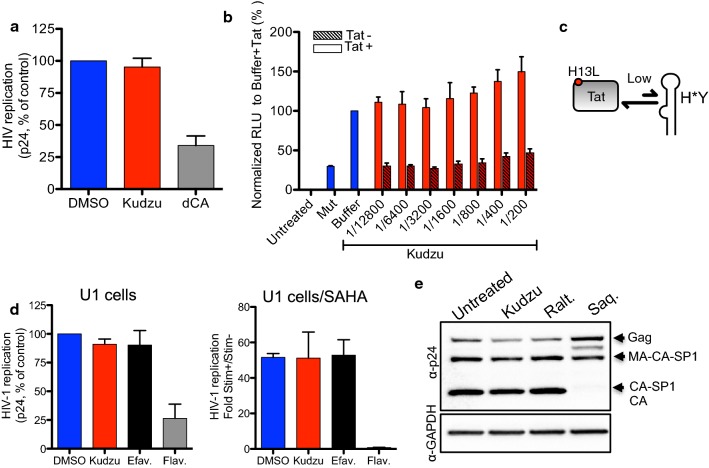
Kudzu does not interfere with HIV-1 late replication events. **a** Kudzu has no activity on HeLa-CD4 cells chronically infected with NL4-3. Kudzu used at 1:400 dilution. Didehydro-Cortistatin A (dCA, a Tat inhibitor, 100 nM) was used as a control. Viral supernatants recovered 72 h post-infection from cells were assayed for their p24 antigen content. Data is the mean ± SD of 2 independent experiments. **b** Kudzu does not impact the transactivation activity of transfected Tat protein in TZM-bl cells. Tat mutated in the basic domain, Tat Mut, and buffer were used as negative controls. Luciferase activity per protein concentration was determined 48 h later. The data represents the mean ± SD of 2 independent experiments. **c** Schematic describing the mutation in Tat protein in U1 cells. **d** Kudzu has no activity on chronically-infected U1 cells before and after stimulation with SAHA (an histone deacetylase inhibitor, 1 μM). Kudzu used at 1:400 dilution. Flavopiridol (Flav., a CDK9 inhibitor, 100 nM) and Efavirenz (Efav., 100 nM) were used as controls. Viral supernatants recovered 72 h post-infection from cells were assayed for their p24 antigen content. Results are the mean ± SD of 3 independent experiments. **e** Kudzu has no activity on maturation and assembly of HIV-1 capsid. HIV-1 capsid originates from a 55 kDa Gag precursor proteolyzed into three folded proteins [matrix (MA), capsid (CA) and nucleocapsid (NC)] and 3 small peptides [spacer peptides 1 and 2 (SP1 and SP2) and p6]. HEK293T cells were transfected with NL4-3 in presence of compounds, and 72 h later, cell lysates were analyzed by western blot with anti-p24 antibody. Kudzu used at the dilution 1:400. Raltegravir (Ralt., 100 nM) and Saquinavir (300 nM) used as controls. The result are representative of 3 independent experiments

We also investigated the ability of Kudzu to inhibit late replication events, i.e., after integration of the viral DNA into the host cell genome. First, we tested the activity of Kudzu on viral transcription by analyzing the viral production from chronically-infected HeLa-CD4 cells for a period of 72 h (Fig. 5a). We used the Tat inhibitor didehydro-Cortistatin A (dCA) as a control [39]. While dCA inhibited viral production from these cells, Kudzu (1:400) showed no activity. We further confirmed this result by measuring the activity of Kudzu during Tat transactivation of the HIV-1 promoter in TZM-bl cells (Fig. 5b). A dominant negative variant of Tat mutated in the basic domain (“Tat Mut”), and unable to transactivate HIV-1 promoter, was used as a negative control. We confirmed that Kudzu had no impact on Tat-mediated transcription of HIV-1 while not altering the viability of TZM-bl cells (Additional file 1: Fig. 2SD right). We also investigated the activity of Kudzu on basal transcription using U1 cells, a model of HIV-1 latency where a H13L mutation in Tat abolishes the activity of the protein (Fig. 5c and d). The controls performed as expected, with a CDK9 inhibitor, Flavopiridol, inhibiting basal transcription, but not Efavirenz (Fig. 5d left). In this case again, Kudzu (1:400) did not alter basal transcription. We observed the same results after stimulating the cells with Suberoylanilide Hydroxamic acid (SAHA) for 24 h, a histone deacetylase inhibitor (Fig. 5d right). Collectively, these data suggest that Kudzu does not interfere with the transcription of HIV-1 genome.

To investigate whether Kudzu could interfere with late events of the viral life cycle, such as Gag expression and maturation, we transfected HEK293T cells with NL4-3 molecular clone in the presence of Kudzu or controls, and measured Gag expression in cell lysates by immunoblot, using an anti-p24 antibody (Fig. 5e). The expression of Gag protein and its maturation were similar in Kudzu (1:400), Raltegravir (100 nM), and untreated samples, while the protease inhibitor, Saquinavir (300 nM), blocked the maturation of Gag into Matrix (MA) and Capsid (CA) proteins. Taken together, our results strongly suggest that Kudzu does not impact the late events of HIV-1 life cycle.

### Individual antiviral activity of the chemical components of Kudzu

**Fig. 6:**
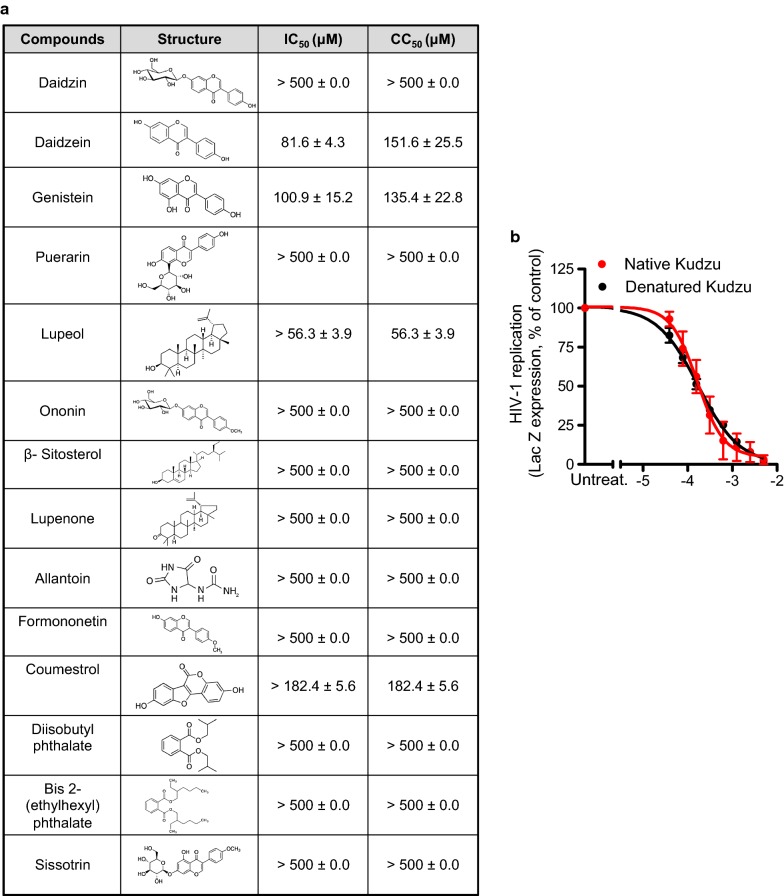
Activity of the different components of Kudzu on acute replication of HIV-1 of X4 tropic virus in HeLa-CD4-LTR-LacZ cells. **a** HeLa-CD4-LTR-LacZ cells were infected with HIV-1 NL4-3 strain in presence of different concentrations of the indicated compounds. β-Gal activity and viability assays were performed 72 h later. Results represent the mean ± SD of 3 independent experiments. **b** The activity of Kudzu does not involve proteins. HeLa-CD4-LTR-LacZ cells were infected with HIV-1 NL4-3 strain in presence of denatured Kudzu by heat or native Kudzu. β-Gal activity was measured 72 h later. Data is the mean ± SD of 4 independent experiments. Untreat.: untreated

The geographic origin, as well as the methods of culture and root extraction, dictate the chemical composition of Kudzu extract. Several reports ascribe more than 40 chemical entities to Kudzu root extract (Additional file 1: Table 1S). In an effort to identify the compound responsible for Kudzu’s activity, we tested its main components and as many as of the commercially available less abundant ones (Fig. 6a). To our surprise, none of the most abundant components presented antiviral activity, including Puerarin (60% of the total isoflavones in Kudzu), Daidzein and Daidzin, which have been previously reported to have anti-microbial activity [25]. In addition, the combination of the highly represented compounds still did not display antiviral activity (data not shown). Moreover, we assessed whether the activity of Kudzu was potentially due to protein traces in the extract. For this, we denatured Kudzu by boiling and tested its activity in infectivity assays using HeLa-CD4-LTR-LacZ cells. Figure 6b shows that the denaturation did not affect the activity of Kudzu. Furthermore, the denatured Kudzu was ran on a denatured electrophoresis gel, transferred to a nitrocellulose membrane, and no proteins were observed after Coomassie blue staining (data not shown). Altogether, these results suggest that Kudzu antiviral activity most likely derives from a component present at a very low concentration or it results from an additive/synergistic effect of several compounds.

### Activity of Kudzu in combination with current ART and activity against viruses resistant to Enfuvirtide

**Fig. 7:**
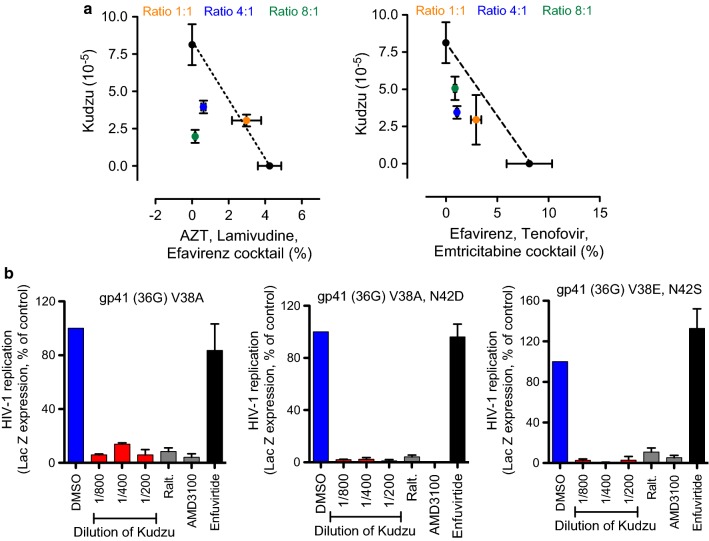
Activity of Kudzu in combination with antiretrovirals. **a** Combination of Kudzu with different cocktails of antiretrovirals prescribed to HIV-1 patients. HeLa-CD4-LTR-LacZ cells were infected with HIV-1 NL4-3 strain in presence of different amounts of Kudzu or the indicated antiretrovirals. β-Gal activity was measured 72 h later. The IC_50_ of the cocktails or Kudzu alone (black) or the mix of both at different ratios (orange, green, and blue) are represented. Results represent the mean ± SD of 3 independent experiments. **b** Activity of Kudzu in HeLa-CD4-LTR-LacZ cells infected with viruses resistant to Enfuvirtide. HeLa-CD4-LTR-LacZ cells were infected in presence of different dilutions of Kudzu. β-Gal activity was measured 72 h later. AMD3100 and Enfuvirtide used at 10 nM and 0.15 μg/ml respectively. Data is the mean ± SD of 2 independent experiments

We assessed the activity of Kudzu in combination with two different cocktails: ATRIPLA^®^ (Efavirenz, Tenofovir and Emtricitabine) as a single tablet regime, and ALE, Combivir (AZT and Lamivudine) combined with Sustiva (Efavirenz). We first determined the IC_50_ of each ARV cocktail and Kudzu alone. Next, Kudzu and the cocktail at their IC_50_ were mixed together at different ratios (Kudzu: cocktail, 1:1, 4:1, 8:1) to obtain an IC_50_ of the mix. The data was plotted as concentration of ARVs cocktails per Kudzu dilution (Fig. 7a). The mixes of Kudzu with ATRIPLA^®^ at the different ratios presented IC_50_ concentrations lower than the trend line (dotted line between Individual IC_50_), which is associated with synergistic activity [40]. With the ALE cocktail, the activity of Kudzu was additive when used at ratio of 1:1, and synergistic when used at doses higher than 1:1.

A number of characterized HIV-1 isolates are resistant to the entry inhibitor Enfuvirtide. We evaluated the susceptibility to Kudzu of three of these viral isolates, which carry mutations in gp41 (gp41 D36G V38E N42S, gp41 D36G V38A and gp41 D36G V38A N42D) [41]. As expected, these viruses were resistant to Enfuvirtide (150 ng/ml). The inhibition by Kudzu was as potent as Raltegravir (100 nM) and AMD3100 (10 nM).

Collectively these results suggest that Kudzu does not compete with current ART, but acts additively/synergistically with ART, rationalizing its use as a complement to current HIV-1 therapy. Kudzu may also be helpful in cases of Enfuvirtide resistance, highlighting its potential to complement HIV-1 salvage therapy.

Importantly, we did not observe development of resistance after passaging the NL4-3 strain every 4 days, onto naïve HeLa-CD4 cells in the presence of sub IC_50_ concentrations over a 54 days period.

## Conclusion

Only a few agents are currently used in the clinic that target the HIV-1 entry process (Enfuvirtide, Maraviroc and Ibalizumab). There is a continuing need for development of novel antiretroviral drugs and regimens in order to address tolerability, long-term safety concerns, the immune dysfunction mediated by HIV and the emergency of drug resistance. The cost of current treatment and accessibility is also a concern to a significant portion of the HIV affected population. Traditional medicine is very popular in many countries, and it has been used to replace ARVs or off-set side effects from antiretroviral medication. Such medicinal plants have not been properly identified and documented probably due to the lack of collaborations between scientists and traditional healers. Kudzu is a safe and very well-known natural product that has been used for hundreds of years. Here we demonstrated its potent activity against the attachment of HIV-1 gp120 to receptors on the host cell membrane, blocking HIV entry into cell lines, primary CD4^+^T cells and macrophages. To date, no small molecule compound has been described that targets this step of the life cycle. Kudzu’s efficacy against HIV-2 is further appreciated given that HIV-2 is naturally resistant to Enfuvirtide, nonnucleoside reverse transcriptase inhibitors, and to some protease inhibitors. Moreover, Kudzu has also been shown to reduce inflammation and oxidative stress, an affliction that is often identified in HIV infected individuals. So far, we have not been able to identify the chemical entity of Kudzu extract that is responsible for the antiretroviral activity. In future studies, we will use a combination of HPLC fractionation and 1H-NMR to identify active compounds. In sum, given Kudzu’s low cost, safety, oral bioavailability, tissue distribution, activity with ART and potent activity against HIV gp120 attachment to host cell, it should be considered as a promising supplement to current HIV therapeutic strategies.

## Methods

### Kudzu

Kudzu (Hawaii pharm LLC), was used in all experiments. Its activity was confirmed with Kudzu from Secrets of the Tribe (Nevada pharm LLC). Both Kudzus are a tincture solution with 33% of Kudzu and 66% of ethanol-glycerol-water.

### Viral replication of HIV-1 in cell lines detected by p24 ELISA

HeLa-CD4-LTR-LacZ cells (5 × 10^3^/well in a 96-well-plate) were first incubated with Kudzu or the indicated controls (Raltegravir: 100 nM, Efavirenz: 100 nM, AMD3100: 10 nM, Enfuvirtide: 1 μg/ml,) then infected overnight (ON) with wild-type HIV-1 isolate NL4-3 (3.2 ng/well). Cells were washed and further cultured in fresh media (DMEM supplemented with 5% Fetal bovine serum (FBS), l-glutamine (292 μg/ml) and antibiotics (100 units/ml penicillin and streptomycin)) containing compounds for another 72 h. HIV-1 replication was assessed by measuring the viral capsid in the supernatant with a p24 ELISA kit (Advances Bioscience Laboratories). Similar protocol was used for the infection of GHOST-CCR5 cells (gift from Dr. Michael Farzan) with JRCSF (21.3 ng/well, NIH AIDS Reagent Program, cat# 394) or YU2 (2.32 ng, NIH AIDS Reagent Program, cat# 1350) viruses. The amount of virus was chosen to obtain similar p24 capsid outputs between the different cell lines.

### Viral replication of HIV-1 in cell lines, revealed by chlorophenol red-β-d-galactopyranoside assay (CPRG assay)

HeLa-CD4-LTR-LacZ cells (5 × 10^3^/well in a 96-well-plate) were first incubated with compounds (Kudzu, Kudzu’s components, indicated ARVs controls, Glycerol, Ethanol, or denatured Kudzu boiled for 10 min at 90 °C). NL4-3 virus (6.6 ng/well) or viruses resistant to Enfuvirtide (gifts from NIH AIDS Reagent Program, cat# 9490 (5.4 ng/well), cat# 9488 (2 ng/well), cat# 9496 (0.6 ng/ml)) were then added for 72 h. Cells were disrupted with lysis buffer (60 mM Na2HPO4, 40 mM NaH2PO4, 10 mM KCl, 10 mM MgSO4, 2.5 mM EDTA, 50 mM ß-mercaptoethanol, 0.125% Nonidet P-40) for 1 h at 4 °C and a quantitative CPRG-based (Boehringer Mannheim) assay was performed per manufacturer’s instructions. The cell extracts were incubated in reaction buffer (0.9 M phosphate buffer [pH 7.4], 9 mM MgCl2, 11 mM ß-mercaptoethanol, 7 mM CPRG) until a red color developed and measured with an LP400 (Becton–Dickinson) plate reader at 572 nm. The amount of virus used for infection aimed to produce a similar OD at approximately 20 min of reaction.

### Viral replication of H1N1, ZIKV Brazil and ADV5 viruses

Influenza A (Virginia/ATCC1/2009 H1N1), Zika virus (PB81, Brazil strain), and adenovirus serotype 5 (ADV5) were produced in MDCK, Vero and HEK293T cells, respectively. Each virus was mixed with the indicated compounds, and added to Hela-CD4-LTR-LacZ cells. Aleuria Aurantia lectin (100 nM), an inhibitor of H1N1 entry, cabozantinib (1 μM), a tyrosine kinase inhibitor known to suppress Zika infection, and heat inactivation of ADV5 for 30 min at 70 °C, were used as positive controls. After 1 h at 37 °C, the inoculum was removed, and the cells further grown in DMEM containing 10% FBS and the indicated compounds. Twenty-four hours after the inoculation, the cells were collected, fixed with PBS containing 2% of paraformaldehyde, permeabilized using PBS containing 2% of saponin, and stained with either the monoclonal mouse anti-H1N1/H2N2 antibody clone C179 (H1N1), or the monoclonal mouse pan anti-flavivirus antibody clone 4G2 (Zika), or the mouse monoclonal anti-adenovirus antibody clone 2/6 (ADV5), followed with an Alexa647-conjugated, anti-mIgG antibody (Jackson ImmunoResearch). The cells were then washed, fixed, and analyzed using flow cytometry (BD Biosciences C6 Accuri and IntelliCyt HyperCyt sampler powered by FlowCyt software). Infection levels were normalized to those of cells infected in the absence of any compound.

### Viral replication of pseudotyped viruses

Hela-CD4-LTR-LacZ cells (1 × 10^5^/in a 6-well-plate) were infected ON with VSV-G-NL4-3 (70.2 ng or 5.6 ng/well) or with HXB2 gp160-NL4-3 (25.4 ng/well) pseudotyped viruses (constructs gifted by Dr. Michael Farzan), in the presence of Kudzu (1:400) or controls (Raltegravir: 200 nM, Efavirenz: 200 nM, AMD3100: 10 nM, Enfuvirtide: 1 μg/ml). Cells were washed and fresh media containing compounds was added for 72 h. The capsid p24 in the supernatant was assessed by ELISA.

### Viral replication of HIV2 CBL-20 strain

TZM-bl cells (4 × 10^4^/well in a 6-well-plate) were incubated with different concentrations of Kudzu or the indicated controls, then infected ON with HIV-2 CBL-20 virus (NIH AIDS Reagent Program, cat# 600, 0.2 ng/well). Media was then removed and fresh media (DMEM supplemented with 5% FBS, l-glutamine (292 μg/ml) and antibiotics (100 units/ml penicillin and streptomycin)) containing compounds were added for an additional 48 h. Luciferase activity was then determined and reported per protein concentration of each sample as previously described [39].

### Viral replication of HIV-1 and SIV in primary human and rhesus macaque CD4^+^T cells

CD4^+^T cells were isolated and expanded as previously described [42]. Briefly, PBMCs were extracted from 3 healthy human donors (purchased from One Blood-Florida) and 3 healthy rhesus macaques (obtained from the Wisconsin National Primate Research Center). Total CD4^+^T cells were isolated using positive selection kit (StemCell Technologies). Cells were expanded with 1 μg/ml of PHA (Sigma Aldrich), 100 U/ml of IL-2 (Roche) and irradiated feeder PBMCs (OneBlood) and cultured in RPMI and human serum for 3 days. Primary human CD4^+^ T cells were treated with DMSO, kudzu or a cocktail of ARVs (AZT: 180 nM, Efavirenz: 100 nM, Raltegravir: 200 nM) then infected with NL4-3 (100 ng). After 24 h, cells were pelleted, DNA extracted, followed by PCR (see below). Primary macaque CD4^+^T cells were treated with DMSO, Kudzu or a cocktail of ARVs (Emtracitabine, Raltegravir and Tenofovir, 200 nM) then infected with SIV_mac_239 (100 ng). SIV replication was assessed 6 days later, by measuring the viral capsid in the supernatant with p27 ELISA (Advances Bioscience Laboratories).

### Viral replication of ADA and 5002M viruses in primary macrophages

Human monocyte-derived macrophages were isolated from elutriated blood PBMCs by Ficoll-Hypaque density gradient centrifugation (GE Healthcare). Monocytes were negatively selected using magnetic particles from EasySep™ Human Monocyte CD14 + without CD16 depletion Enrichment kit (Stem Cell Technologies). Monocytes were differentiated in DMEM (Gibco) supplemented with 10% heat inactivated human serum (Sera Care Life Sciences), 2 mM L–glutamine (Gibco), 10 μg/ml gentamicin (Sigma–Aldrich) and 10 ng⁄ml MCSF (R&D System) and cultured for 7 days at 37ºC with 5% CO_2_. Macrophages were then seeded onto 48-well plate (1 × 10^5^ cells/well), infected with 1 × 10^6^ CPMs of macrophage-tropic strains ADA or 5002M in the presence of kudzu (1:100) or Enfuvirtide (5 μg/ml). Viral inoculum was removed, and media replaced with fresh compounds 24 h post-infection. Compounds were added every day to the media, and half media was replaced every 2 days. An aliquot of the supernatant was harvested every day for 6 days, for capsid p24 quantification (ClonTech).

### Viral replication of HCV, assessed by immunohistochemistry

Huh 7.5 cells were infected with HCV JC1 virus and Kudzu (at the dilution 1:200) was subsequently added. A blank (without compounds, “untreated”) or 2′-C-methyladenosine (10 μM) [43] was used as control. After 48 h, cell supernatants/lysates were harvested, cell lysates prepared with 2 cycles of freeze thawing, and stored (− 80 °C) for infection of naïve cells for titration. Naïve Huh-7.5 cells (cultured in 96-well plates) were inoculated with the supernatant/lysates for limiting dilution virus titration assay. After 48 h, cells were washed, fixed in methanol, and then probed for NS5A expression using the 9E10 monoclonal antibody. TCID_50_ was calculated as previously described [44].

### Latently infected cells

NL4-3 latently infected HeLa-CD4 cells (2.5 × 10^5^/well) and U1 cells (5 × 10^5^/well) were treated with compounds for 72 h, and p24 in the supernatant was assessed by ELISA. HeLa-CD4 cells were treated with 1:400 dilution of Kudzu or 100 nM of dCA. U1 cells were treated with 1:400 dilution of Kudzu, 100 nM of Efavirenz or 100 nM of Flavopiridol. U1 cells reactivation was performed with 1 μM of SAHA for 24 h.

### Time-of-addition experiment

HeLa-CD4-LTR-LacZ cells (5 × 10^3^/well in a 96-well-plate) were infected with NL4-3 (6.6 ng/well). Kudzu and ARVs controls were added to the infected cells, at the indicated concentrations, at time 0, 1, 2, 3, 4, 5, and 6 h post-infection. β-Gal activity was measured 72 h later.

### Attachment assay in TZM-bl cells

TZM-bl cells (2 × 10^4^) were seeded in a 96-well plate. Next day, cells were incubated for 1 h at 4 °C. In parallel, compounds (Kudzu: 1:400–1:100, Heparin: 1 mg/ml, Enfuvirtide: 1 μg/ml and AMD3100: 10 nM) were mixed with NL4-3 (16.6 ng/well) or HIV-2 CBL-20 (0.8 ng/well) for 1 h at 4 °C. Compounds with virus were then added to the cells and incubated at 4 °C for 3 h. Cells were washed twice with ice cold PBS and incubated with fresh media for 72 h at 37 °C, 5% CO_2_. The infection was assessed by measuring the luminescence with Bright Glo (Promega) according to the manufacturer’s protocol.

### Transactivation assay in TZM-bl cells

Cells were seeded at 2 × 10^6^ in a 10 cm^2^ tissue culture dish and transfected with 5 μg of the constructs expressing Tat (PGK-Flag-Tat) or Tat Mut (PGK-Flag-Tat Mut) driven by the murine phosphoglycerate kinase-1 (PGK) promoter, with TransIT-LT1 transfection reagent (Mirus Bio LLC) according to the manufacturer’s protocol. The cells were split 24 h post transfection and treated with Kudzu at different concentrations. Luciferase activity per protein concentration of each sample was determined 48 h later as previously described [39].

### Quantification of early and late reverse transcription product and provirus integration

#### In HeLa-CD4-LTR-LacZ cells

Cells were plated at 1 × 10^5^ cells per well in a six-well plate. Twenty-four hours later, cells were infected with NL4-3 virus (66 ng) in the presence of DMSO, Kudzu (1:200), Efavirenz (200 nM), Saquinavir (200 nM), Raltegravir (200 nM) and AMD3100 (10 nM). At 10 h and 24 h post-infection, genomic DNA was prepared and early and late viral DNA products and the integrated proviruses were quantified as previously described [45].

#### In primary CD4^+^T lymphocyte cells

Cell pellets were digested at 133 μl/million cells with lysis buffer (10 mM Tris–HCL, 50 nM KCl, 400 µg/ml proteinase K (Invitrogen) to extract DNA. Total HIV or SIV DNA content was quantified by PCR amplification with Taq polymerase, 1X Taq Buffer (Invitrogen), MgCl_2_, dNTPs, HIV/SIV and CD3 primers for 12 cycles. The second amplification involved a nested PCR using 1:10 dilution of amplification product, HIV/SIV and CD3 primers, SsoAdvanced Universal Probe Supermix (Biorad) using qPCR. A standard curve was established using ACH2 cell lysates (ATCC) that contain a single copy of HIV per cell or SIV_mac_239 plasmid. Primers used are listed in Additional file 1: Table S2.

### gp120-CD4 interaction by ELISA

Recombinant YU2 gp120 protein (Immune Tech, cat# IT-001-0027p, 1.5 μg/ml) was coated onto a 96-well ELISA plate ON at 4 °C. Next day, wells were washed twice with 150 μl of PBS supplemented with Tween 0.05%, and saturated with 5% milk for 1 h at 37 °C. CD4-Ig (gift from Dr. Michael Farzan, 5 μg/ml) in presence or not of increasing concentrations of Kudzu was then added and the plate was incubated at 37 °C for 1 h. Secondary anti-human antibodies were then added at the dilution 1:5000 for 1 h at 37 °C. Then, 50 μl of TMB (Immunochemistry) was added at room temperature and the reaction was stopped with TMB stop solution. The absorbance was read at 450 nm. Several washes were performed after each step except after saturation. Emtracitabine (500 μM), soluble recombinant YU2 gp120 protein (Immune Tech, cat# IT-001-0027p) and soluble mouse CD4 (gift from Dr. Michael Farzan) were used as controls in the assay.

### Expression of CD4 and CXCR4 in HeLa-CD4-LTR-LacZ cells analyzed by flow cytometry

Cells were seeded at 1 × 10^6^ in a 6-well plate. The next day, compounds (Kudzu 1:400, PMA 100 nM or buffer) were added. Cells were then collected at different time points, stained with PE/Cy7 anti-human CD184 (for CXCR4, Biolegend) or PE antihuman CD4 (Biolegend) and fixed with 2% formaldehyde in PBS. Cells were analyzed on a LSRII flow cytometer (BD Bioscience).

### Expression of CCR5 in Ghost-CCR5 cells analyzed by flow cytometry

Cells were seeded at 1 × 10^6^ per 6-well plate. The following day, compounds (Kudzu 1:400 and 1:200) were added. Cells were then collected at 6 h post treatment, stained with PE anti-human CD195 (Biolegend) or PE control (Biolegend). Flow cytometry was afterwards performed (BD Biosciences C6 Accuri and IntelliCyt HyperCyt sampler powered by FlowCyt software).

### Fusion of HeLa-CD4-LTR-LacZ cells with HL2/3 cells

Similar protocol to [27] was performed. Briefly, HL2/3 cells (gift from AIDSreagent program), expressing the HXB2 envelope, Tat, Gag, Rev, and Nef proteins, were co-cultured with HeLa-CD4-LTR-LacZ cells at 1:1 density ratio (2.5 × 10^4^ cells/well in a 96-well-plate) for 48 h in presence of different compounds (Kudzu: 1:400 and 1:200, Raltegravir: 100 nM, Emtricitabine: 100 nM, Enfuvirtide: 1 μg/ml). Upon fusion of the two cell lines, Tat protein from HL2/3 cells activates β-gal expression, measured by a CPRG assay.

### Shedding of gp120 from transfected gp160

Performed as previously reported [35]. Briefly, HEK293T cells were transfected with the constructs JR-FL gp160Δtail (1.5 μg) and pCMV Rev (0.5 μg) (gifts from Dr. Michael Farzan) for 48 h with TransIT-2020 transfection reagent (Mirus Bio LLC) according to the manufacturer’s protocol. JR-FL gp160Δtail was used to achieve higher envelope trimer expression [46]. CD4-Ig (45 μg, a gift from Dr. Michael Farzan) or Kudzu (1:400 dilution) were incubated with cells for 4 h. Cells were then washed several times with PBS 10 mM EDTA and lysed with RIPA buffer supplemented with a cocktail of protease inhibitor (Roche). Protein concentration was determined by Bradford assay. The cell lysates were analyzed on a reducing gel 4–20% gradient gel (Biorad) and subsequently blotted onto a nitrocellulose membrane (Biorad). gp120 content was determined by immunoblotting using a gp120 serum at 1:500 dilution (a gift from Dr. Michael Farzan) and goat anti-human antibody (1:5000, Santa Cruz).

### Shedding of gp120 from NL4-3 virus

Kudzu and CD4-Ig (gift from Dr. Michael Farzan, 30 μg) were incubated with NL4-3 (44.8 ng) for 6 h. Samples were then filtered through 300 kDa column (Vivaspin, Sartorius). A fraction of the flow trough was analyzed by immunoblot as described above.

### Maturation of HIV-1 capsid

HEK293T cells were transfected with pNL4-3 encoding plasmid (12.5 μg) with TransIT-2020 transfection reagent (Mirus Bio LLC) according to the manufacturer’s protocol. Six hours later, cells were washed, trypsinized and split between the conditions tested. Cells were treated with either buffer, Kudzu (1:400), Raltegravir (100 nM), Saquinavir (300 nM) and incubated at 37 °C for 72 h. Next, cells were lysed in RIPA buffer supplemented with a cocktail of protease inhibitors (Roche) and analyzed by immunoblotting with anti-p24 antibody (1:2500, NIH AIDS Reagent Program cat# 3537). Anti-GAPDH antibody (1:500, Santa cruz) was used as loading control.

### Assessment of Kudzu’s cytotoxicity

MTT (3-[4,5-dimethylthiazol-2-yl]-2,5-diphenyltetrazolium bromide) assay (ATCC) or cell titer Glo luminescent cell viability (Promega) was performed in the presence of increasing concentrations of Kudzu or Kudzu components according to the manufacturer’s protocol.

## **Additional file**

**Additional file 1: Table 1S.** Published Components of Kudzu from China. **Figure 1S.** Cytotoxicity of the cells used. **Figure 2S.** Activity of Kudzu’s vehicles (glycerol and Ethanol) and ARVs in acute infection of HeLa-CD4-LTR-LacZ cells with an X4 tropic virus. **Figure 3S.** Kudzu does not alter CD4, CXCR4 and CCR5 cell membrane expression. **Table 2S.** Table of primers/probes used.
