# Supplementary material for: Potent suppression of HIV-1 cell attachment by Kudzu root extract
Source: Retrovirology. 2018 Sep 20;15:64. doi: 10.1186/s12977-018-0446-x (PMC6149077; doi:10.1186/s12977-018-0446-x)
Supplement: Supplementary file 1 — Additional file 1: Table 1S. Published Components of Kudzu from China. Figure 1S. Cytotoxicity of the cells used. Figure 2S. Activity of Kudzu’s vehicles (glycerol and Ethanol) and ARVs in acute infection of HeLa-CD4-LTR-LacZ cells with an X4 tropic virus. Figure 3S. Kudzu does not alter CD4, CXCR4 and CCR5 cell membrane expression. Table 2S. Table of primers/probes used. [file 12977_2018_446_MOESM1_ESM.pdf]

PUERARIA LOBATA (ROOT)			
**References**	Liu *et al*., 2015	Li *et al*., 2010	Du *et al*., 2010
**Origin**	Crude extract (China)		
**Techniques**	Dual high-Resolution alpha glycosidase inhibition and radical scavenging profiling combined with hyphenated HPLC-HRMS-SPE-NMR	HPLC followed by NMR	Microwave-assisted extraction and ultra high performance liquid chromatography coupled with diode array detection and time-of-flight mass spectrometry
**Compounds**	Puerarin	-	Puerarin
	3′-hydroxypuerarin	-	3'-hydroxypuerarin
	3′-methoxypuerarin	-	3′-methoxypuerarin
	-	-	Puerarin-4'-O-glucoside
	-	-	Puerarin-3'methyoxy-4'-O-glucoside
	-	-	6''-O-xylosylpuerarin
	6″-*O*-α-d-apiofuranosylpuerarin	-	-
	Daidzin		
	-	-	Daidzin-4',7-O-glucoside
	6″-*O*-malonyldaidzin	-	-
	3′-methoxydaidzin	-	-
	Daidzein		
	Daidzein 8-*C*-[β-d xylopyranosyl-(1→6)]-β-d-glucopyranoside	-	-
	daidzein 4′-β-d-glucopyranoside	-	-
	-	3'-methoxy daidzein	-
	-	3'-hydroxy daidzein	-
	-	-	Isodaidzein
	Genistein	-	Genistein
	Genistein 8-*C*-α-d-apiosyl-(1→6)-β-d-glucoside	-	-
	Genistein 8-*C*-β-d-glucoside	-	-
	Genistin		
	6″-*O*-malonylgenistin	-	-
	Ononin		
	6″-*O*-malonylononin	-	-
	-	8-methoxy ononin	-
	Formononetin		
	Sissotrin	Sissotorin	-
	-	-	Biochanin A
	Biochanin A 7-*O*-β-d-glucoside-6″-*O*-malonate	-	-
	-	β-Sitosterol palmitate	-
	-	β-Sitosterol	-
	-	Lupeol	-
	-	Lupeone	-
	-	Puerarol	-
	-	Diisobutyl phthalate	-
	-	Bis (2-ethylhexyl) phthalate	-
	-	Sophoracoumestan A	-
	-	Coumestrol	-
	-	Allantion	-
	-	(-)-puerol B 2-O-glucopyranoside	-
	Puerol B 2-*O*-β-d-glucopyranoside	-	-
	-	(6S, 9R)-roseoside	-
	-	Sucrose	-
	-	-	Pseudobaptigenin
	-	-	6''-O-Apiosyl
	-	-	Sophoraside A
	-	-	Mirificin

Table 1S. Published Components of Kudzu from China.

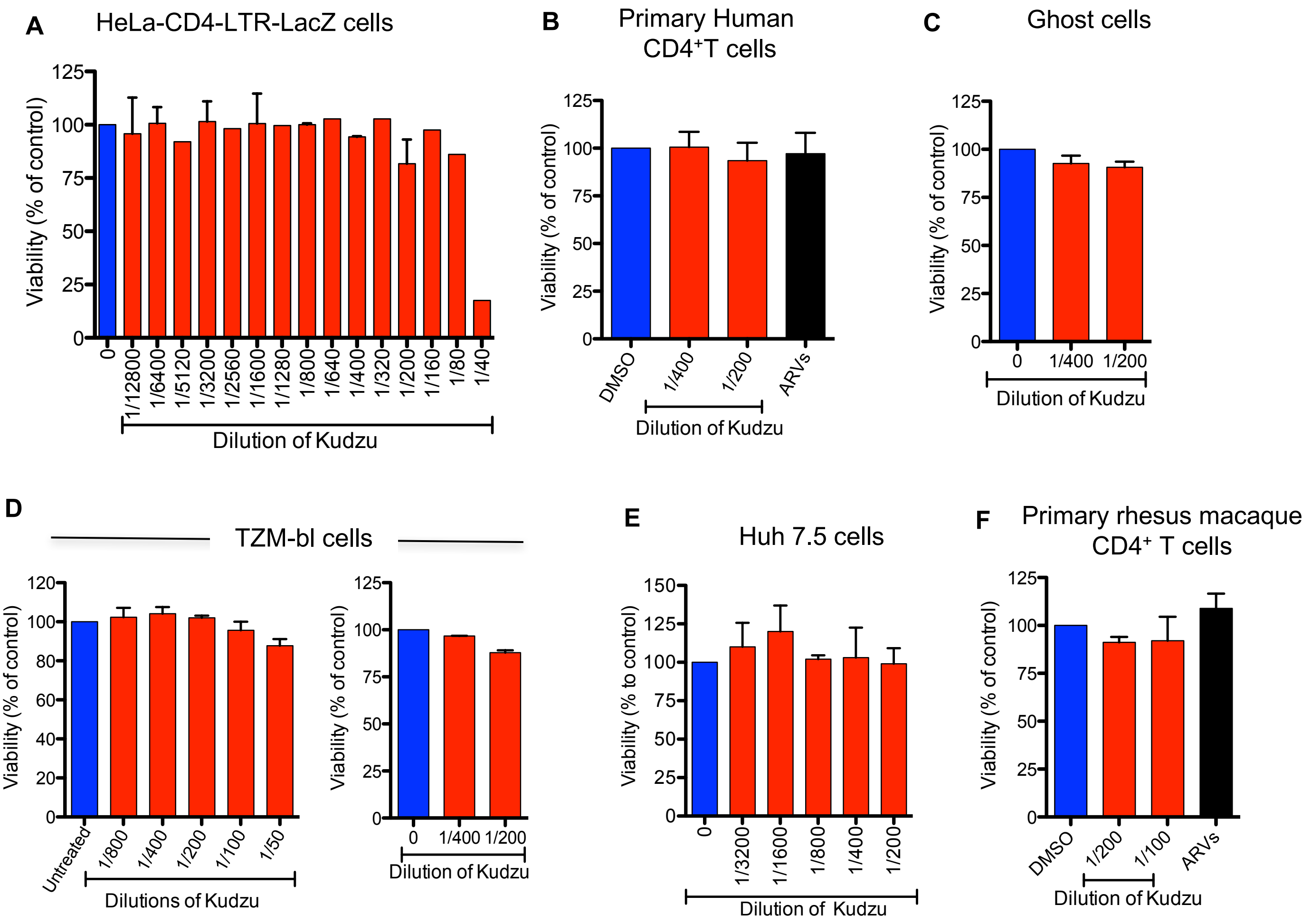

Figure 1S. Cytotoxicity of the cells used. Viability assays performed in (**A**) HeLa-CD4-LTR-LacZ cells 72 h post-treatment in presence of different concentrations of Kudzu. (**B**) HIV-infected primary human CD4$^+$T cells cells 24 h post infection. (**C**) Ghost cells 72 h treatment with Kudzu. ARVs: antiretrovirals (Raltegravir 200 nM, Efavirenz 100 nM, AZT 180 nM), (**D**) TZM-bl cells 3 (on the left) or 72 h (on the right) post-treatment. (**E**) Huh 7.5 cells 72 h post-treatment. (**F**) SIV-infected primary rhesus macaque CD4$^+$T cells 6 days post infection. Results represent average from infection of primary macaque CD4$^+$T cells from three independent rhesus macaques. ARVs: antiretrovirals (Emtracitabine, Raltegravir, Tenofovir, 200 nM). Results represent the mean ± SD of 3 independent experiments for **A**, **B** and **F**, and 2 independent experiments for **C**, **D** and **E.**

A

Class of ARVs		Compound	IC_{50}
		Kudzu (dilution)	1:5263 ± 6.3x10^{-5}
CLASS OF ARVs	Entry inhibitors	Enfuvirtide (nM)	2.3 ± 0.3
		AMD3100 (nM)	0.7 ± 0.1
	Integrase inhibitor	Raltegravir (nM)	4.5 ± 0.6
	Reverse transcriptase inhibitors	Lamivudine (nM)	125.6 ± 2.3
		Efavirenz (nM)	0.5 ± 0.1
		Emtracitabine (nM)	12.0 ± 3.9
		AZT (nM)	4.0 ± 0.7

B

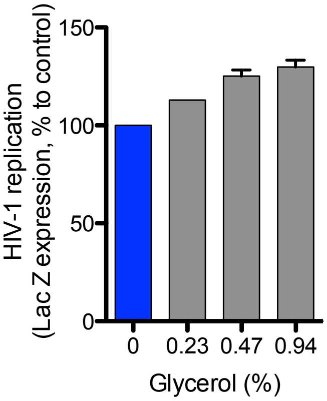

C

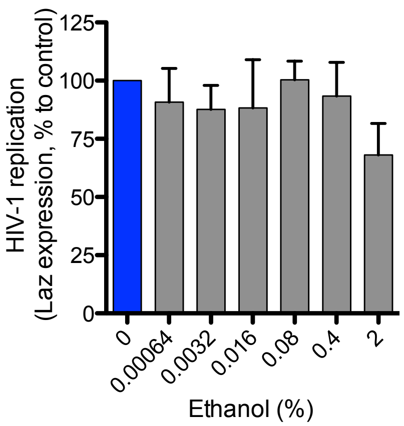

Figure 2S. Activity of Kudzu's vehicles (glycerol and Ethanol) and ARVs in acute infection of HeLa-CD4-LTR-LacZ cells with an X4 tropic virus. (**A**) Table comparing the mean of the IC_{50} ± SD of Kudzu activity and of different potent antiretrovirals (ARVs) against acute infection of HeLa-CD4-LTR-LacZ cells with NL4-3 strain. β-Gal activity was measured 72 later. Shown is mean ± SD of 2 to 5 independent experiments. (**B,C**) HeLa-CD4-LTR-LacZ cells were infected with HIV-1 NL4-3 strain in the presence of different dilutions of Glycerol or Ethanol. β-Gal activity was measured 72 h later. The mean ± SD of 2 independent experiments is represented for the vehicle glycerol condition. The mean ± SEM of 3 independent experiments is shown for the vehicle Ethanol.

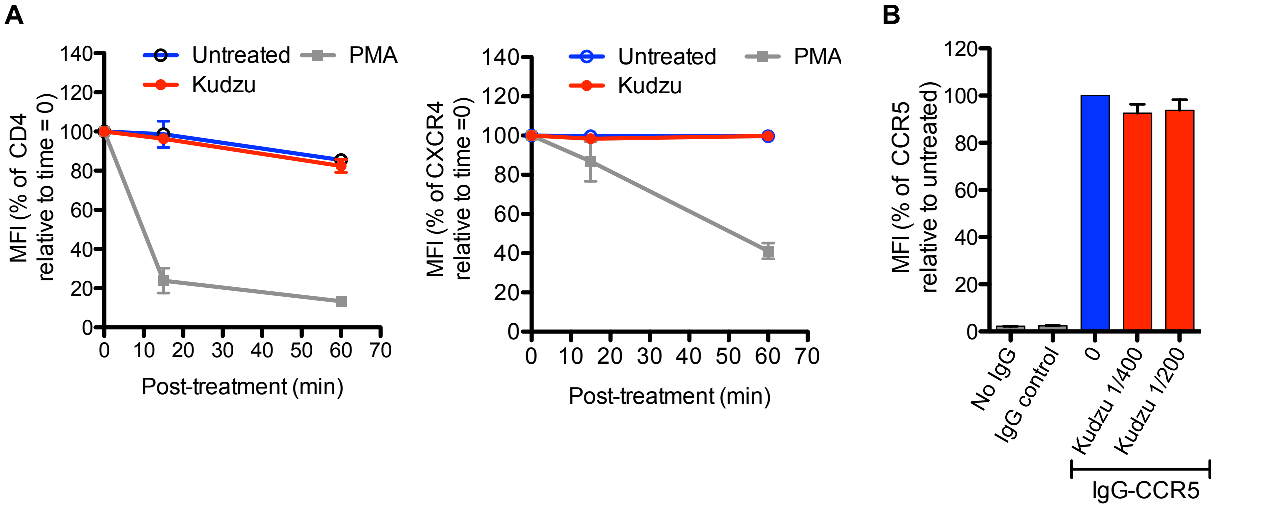

Figure 3S. Kudzu does not alter CD4, CXCR4 and CCR5 cell membrane expression. (**A**) Expression of CD4 and CXCR4 on HeLa-CD4-LTR-LacZ cells in the presence of Kudzu at the dilution 1:400 was detected by FACS. The phorbol ester phorbol myristate acetate (PMA) was used as a control. Results represent the mean ± SD of 2 independent experiments. (**B**) Expression of CCR5 on GHOST-CCR5 cells in the presence of Kudzu at the dilution 1:400 and 1:200 was detected by FACS after 6 h of incubation. Shown is the mean ± SD of 2 independent experiments.

Primer/Probe	Sequence (5’-3’)
CD3OUT5	ACTGACATGGAACAGGGGAAG
CD3OUT3	CCAGCTCTGAAGTAGGGAACATAT
CD3IN5	GGCTATCATTCTTCTTCAAGGT
CD3IN3	CCTCTCTTCAGCCATTTAAGTA
CD3 Taq	LC640AGCAGAGAACAGTTAAGAGCCTCCAT-BBQ
HIV L1	ATGCCACGTAAGCGAAACTCTGGGTCTCTCTDGTTAGAC
HIV R1	CCATCTCTCTCCTTCTAGC
HIV L2	ATGCCACGTAAGCGAAACT
HIV R2	CTGAGGGATCTCTAGTTACC
HIV Taq	LC640-CACTCAAGGCAAGCTTTATTGAGGC-BBQ
SGAG21	GTCTGCGTCATPTGGTGCATTC
SGAG22	CACTAGKTGTCTCTGACTATPTGTTTG
SIV TaqMan	CTTCPTCAGTKTGTTTCACTTTCTCTTCTGCG-(BHQ™1)

Table 2S. Table of primers/probes used.
